# A Treatise on the Causes and Consequences of Habitual Constipation

**Published:** 1840-04-01

**Authors:** 


### A Treatise on the Causes and Consequences of Habitual Constipation. By *John Burne,* M.D. F.R.C.P. Physician to the Westminster Hospital, &c. Octavo, pp. 257. London: Longman and Co. 1840.

This is a bold attempt. For although Dr. Burne says that "the object of this treatise is to direct attention to habitual constipation as a *very frequent cause* of local and general disorder and disease"—yet the catalogue is so extended, and the above cause so prominently brought forward, that nine-tenths of our nosology would appear to be based on "habitual constipation." However, we shall endeavour to present our readers with the chief features of the work itself, instead of indulging in much criticism.

Chap. I. Is entirely physiological, and requires no notice in a medical journal.

Chap. 2. Mentions some of the inconveniences of constipation.

"Not only does it indispose the mind to exertion and the body to exercise, but it casts a gloom over the spirits, and is productive of that general discomfort which ruffles the temper and embitters the ordinary enjoyments of life." 16.

The excrement, he observes, in its natural state, is consistent, soft, homogeneous, cohesive;—whereas, if long retained, it becomes hard, knotty, friable, and dry—" hence, then, an abundant source of impurity to the blood." Irritation of the colon, by the fæces being too long retained, extends, by sympathy, to the liver and stomach, " and by disturbing their function, impairs digestion."

" From impaired digestion, there must proceed impure chyle, from impure chyle impure blood; and if impaired digestion, produced and prolonged by habitual constipation, should endure, not for months only but for years, how can we wonder that the whole mass of the blood should become corrupt, or that the solids derived from that blood should be corrupt also? How can we wonder that eruptions should disfigure the body, or that untractable or malignant disease should break forth and shorten life? Can we hesitate then to believe that habitual constipation may be and is one frequent souice of general disorder and of local disease?" 18.

Our readers will be inclined to think that this is one of the most easy and accommodating theories that ever was invented. Seven cases are related in illustration. It may be useful to glance at some of these.

*Case* 1. Was a female, aged 43, who complained of head-ache, flushings, acidity, flatulence, constipation. After stating these symptoms, she shewed Dr. Burne a tumor in the breast, which he considered to be " indolent scirrhus."

" She was put on a course of alterative stomachic aperient medicine, with a view to regulate the bowels, and improve the general health; but without any special reference to the tumour. She continued under treatment four months, got quite well, and was stronger and better than she had been for some years." 16.

The tumour in the breast had disappeared. Our readers will determine for themselves, whether the removal of the tumour, and of the constitutional symptoms abovementioned, was effected by the mere removal of *constipation*, or by the remedies employed for this purpose, but acting on the whole circle of the chylopoietic viscera.

*Case* 2. Mrs. C. aged 40, had had constipated bowels for about a year—menstruation regular. Had a tumour in the left breast, as large as an egg, with darting pain and tenderness—there was swelling of the arm to the elbow. Her stomach was disordered, and uneasy after food—sometimes sick even to vomiting—appetite bad—emaciation. Dr. B. suspected scirrhus of the pylorus.

" A variety of aperients was tried from time to time; those which agreed best and proved efficacious, were the *infusion and tincture of senna* with *créosote*, and pills composed of the *compound extract of colocynth, opium,* and a little *calomel.* The créasote at all times tended to appease the stomach, and caused aperients to agree where otherwise they would have been rejected. Opium was essential in the treatment; and much good resulted from a mild though prolonged course of mercury, the doses being of *calomel half a grain, opium a quarter of a grain,* taken twice a day after meals. When the gums became sore the mercury was suspended for a few days. In this manner both the calomel and the opium, as also the aperients, were persevered in for many months with great improvement in the health, and the very unexpected subsidence, and ultimately the almost entire dissipation of the tumour in the breast. *Belladonna* in the form of plaster was applied with decided benefit." 21.

Whether a constitutional course of calomel and opium removed the tumour of the breast, and the suspected scirrhus of the pylorus, merely by relieving constipation of the bowels, we leave it to our brethren to determine. We may observe, however, that the treatment of these cases is precisely that which any medical practitioner would employ, who viewed the constipation as merely one of the symptoms in the group, and not the cause of the others. If the constipation were the source of the other phenomena, a course of enemata—of electuary of senna, of sulphur, manna, or other mild aperients, would have been quite sufficient for the purpose.

We shall be able to notice only one more case in this chapter.

*Case* 3. "*Neuralgia from habitual Constipation.*"

Mrs. G., aged 31, had suffered neuralgic twitches in the neck for some weeks. These attacks were periodical, but uncertain in their returns.

"During the paroxysms, the sensibility of the spot, before described, was so heightened that she could not suffer the air to blow upon it, and the slightest touch gave excruciating pain. She had *risings in the throat,* was subject to heats and flushings, to heaviness of the head and other ailments, which it is unnecessary here to detail. Habitually, she was very costive, seldom having dejections without the aid of medicine.

She was instructed to take *five grains of blue pill every night, and the carbonate and sulphate of magnesia with the compound decoction of aloes twice a day.* These medicines kept the bowels freely open; and in the course of five days the neuralgic pains had abated very much, only one paroxysm having occurred during this period. The same treatment being continued, with the addition of *one grain* of the *sulphate of zinc* twice a day, the pains as well as her other complaints, were soon removed entirely." 25.

We submit that, in this case, there is not the slightest proof that "Constipation" was the *cause* of the neuralgia. Why did Dr. B. exhibit blue pill, and ultimately zinc, for simple constipation? It is pretty evident that this female was hysterical, and the active treatment pursued was calculated to rouse all the abdominal and even the pelvic organs into activity of function, and thus cure the complaint. We commend the practice, but we demur to the theory. Other cases where epilepsy, spasms, trismus, &c. are supposed to have arisen from constipation, are given by the Author.

Chap. 3. Treats of the consequences of constipation on the general health, under two heads or stages.

*Stage the first.*—Young females suffer from these consequences more than the older classes.

"Constipation persisting, there soon manifest themselves the primary signs of alvine difficulty; namely, drowsiness and heaviness of the head. Then will be seen in many a darkness under the eyes, while the face is leuco-phlegmatic; or, if the temperament is sanguine, there will be an extraordinary flush of apparent health. To the heaviness of the head is, by and by, superadded pain of various character and situation; it may be an aching, a beating, or a throbbing pain in the forehead or temples, or over one eye, with a sense of great weight or giddinees. Flushings of the face occur, and transient heats over the whole body, though the feet are, at the same time cold. The drowsiness increases; they find it difficult to rouse themselves to any kind of exertion. On going to bed they fall instantly into a sound sleep, which proves heavy but not refreshing; for, on awaking in the morning, they feel tired, unwilling to leave the bed, and if they do not get up at once, sleep quickly overcomes them only to increase the sense of fatigue when they awake again. Menstruation will, in some, continue regular in the midst of this disorder, particularly in constitutions of a rigid fibre; while, in those of a lax fibre, it is often deficient or suppressed; and, in those of a sanguine temperament, abundant even to menorrhagia. Leucorrhœa seldom fails to supervene. The urinary secretion is apt to be copious and colourless; and flatulence in various degrees attends, although the digestion is not much, if at all, impaired." 35.

Those who have attentively watched the *habits* of civilized society—and especially of young females, will be at no loss to assign numerous *other causes* for the train of symptoms above enumerated, than mere constipation of the bowels. The sedentary habits, the late hours, the over-working of the brain, the improper food, &c. &c. leaving aside the tight stays, are amply sufficient to induce the phenomena detailed in the foregoing extract—and constipation into the bargain. Three cases are related in illustration of this first stage of constipation; but they present no satisfactory evidence of the truth of Dr. Burne's theory—though the practice pursued is entirely unobjectionable.

*Second Stage.*—Here the consequences of constipation become formidable indeed; and if many of the sedentary folks of this country, male as well as female, peruse the following passage, they will greatly promote the sale and increase the price of jalap, rhubarb, and castor-oil.

"The determination of blood to the head which, in the first stage produces drowsiness by day and heavy sleep by night, now gradually increasing with the prolonged existence of the cause, interferes at length with the functions of the brain. The mind becomes irritable, apprehensive; noises jar, distract the brain, and strong light overpowers the eye, while at the same time the delicate sensibility of these organs is dulled by the cerebral congestion, and the senses, though morbidly alive to great impressions, are no longer adapted to acute and nice perception. There is, in addition, pain in the head generally, greater in a particular part, as on one side; often of a distracting character, giving sometimes the sensation of the brain opening and shutting; and sooner or later the person falls down in an apoplectic fit. I have known many of these cases, some occurring in persons of a florid complexion and gross habit of body; some in individuals pale and thin; but, in all, there has existed for years the common cause, an obstinate and difficult state of the bowels." 40.

Nine cases are detailed in illustration. Among these are fatal apoplexy, head-aches, indigestion, hypochondriasis, leucorrhœa, prolapsus ani, hemicrania, irregular menstruation, &c. Case 12 in this chapter is very unsatisfactory. A female, aged 55, had been costive almost all her life. She was corpulent and florid. She had "distracting pain in the head, with sense of fulness, and flushings of the face." She fell down in a fit of apoplexy, and died.

Now as there was no *post-mortem* examination, who can say what was the cause of the apoplexy? An organic disease may have been going on for a dozen years; and yet the case is, without hesitation, headed thus: "Apoplexy from Constipation." Surely this is not the kind of evidence that ought to be brought forward by a learned, talented, and experienced physician, as Dr. Burne is.

Chap. IV. "*Sick Head-ache; or Gastro-Hepatic Irritation, Sympathetic of Habitual Constipation.*"

"An attack of sick-headache comes on for the most part suddenly. A lady, having gone to bed as well as usual, will awake in the morning with a sense of great weight in the head and pain in one temple, or over one eye; or, she may be seized with these symptoms at any period of the day; in which case she is obliged to retire to bed. After the pain has continued a few hours she grows sick, then vomits, throwing up quantities of greenish yellow bile and mucus. The vomiting recurs at intervals and the stomach remains irritable, ejecting every thing and resisting the influence of medicines of known efficacy in other cases of gastric irritation, till, at the expiration of twenty-four hours, the sickness and vomiting diminish and aperient medicines are retained, *which in due time act upon the bowels, when the headache, sickness, and vomiting pass away.* The individual soon regains her usual state of health, which continues without any material interruption till she is overtaken by another attack." 49.

The description here is faithful enough; but the sentence which we have marked in italics, though very favourable to the author's theory, is positively incorrect. The head-ache and sickness, in nine cases out of ten, subside *entirely*, at the end of 16—20—or 24 hours, before any action takes place in the bowels. Almost every person who commits a debauch, to which he is not accustomed, or drinks bad wine, will awake with this sick head-ache, and it will run its course, and disappear, whether he takes opening medicine or not. The periodical sick head-aches, to which so many females are subject, have much obscurity in their nature—and although systematic aperients are essential to their removal, we seldom find them sufficient, unless combined with tonics and alteratives. We know many people who have them, and whose bowels are perfectly free from constipation. They have often appeared to us to be connected with malaria—latent gout—irregular menstruation. We see no objection to the following therapeutic measures.

"When a paroxysm is fairly set in, medicine will be of little avail; the head-ache and vomiting will persist in spite of *creasote, opium, hydrocyanic acid, effervescing saline aperients,* and the like; either and all of which will generally be ejected from the stomach, and the symptoms continue their usual period of from 24 to 72 hours. It is of importance, however, to bring about a free action of the bowels as soon as possible, to which end *colocynth, calomel,* and *opium* may be given. The cathartic pills should soon be followed by a *senna and salts draught*, which, if rejected, may nevertheless, as also the pills, be repeated once in eight hours till they have operated freely. Where the constipation is very great, the action of the above medicines should be facilitated by lavements." 55.

Dr. B. depends mainly on a regular course of aperients in the intervals, by way of prevention. We have found arsenic and quinine sometimes necessary. A course of taraxacum is often useful.

CHAP. V. "*On Diseases of the Stomach, as Consequences of Habitual Constipation.*

The catalogue in this chapter comprises indigestion—gastrodynia—pyrosis—"subacuto-chronic" inflammation and organic disease of the stomach.

The following passage is reasonable.

"I do not advance the opinion that habitual constipation is the exclusive cause of the above affections, but that it is the most frequent: a point I urge the more, that, in the treatment of these affections, remedies may be directed to the relief and removal of the constipation, as an essential and chief means of relieving and removing the disorders of the stomach. Not that, in the majority of instances, attention directed only to the relief of the constipation will suffice and supersede all other treatment; for effects, when of long standing, will often persist independent of the original cause, and require means specially adapted to them. Where, however, the cases are recent, the gastric symptoms will yield to, and be entirely removed by, attention to the bowels: as will be shown by instances to be adduced." 58.

Dr. B. excepts "indigestion" from the diseases "most frequently" produced by constipation. And well he might. For a great number of those who labour under its most violent forms, would tell Dr. Burne, that, the only ease they have is, when their stomachs are empty and their bowels firmly constipated.

The other complaints above enumerated, are lightly touched upon, and traced, in great measure at least, to constipation. Wherever torpor of the bowels can be discovered among the symptoms of a case, that phenomenon is singled out as the original mischief-maker. Doubtless it is often to blame—or rather its causes; but a certain personage is seldom so black as he is painted. The article on pyrosis, however, is one of the best in the work, though tinctured with the favourite hypothesis of the author.

The following passages will convey a good idea of Dr. Burne's opinions respecting the nature and causes of this troublesome complaint.

"The history of cases of pyrosis, traced through their several stages from the first symptoms of disordered stomach, will, I think, show that, when pain in the stomach occurs and continues to occur from the presence of food even in moderate or small quantities, there is not merely a heightened organic sensibility of the stomach depending on irritation, but on a subacute inflammation." 63.

Dr. Burne does not believe that the fluid, in pyrosis, comes from the fauces and œsophagus.

"In all cases where the stomach labours after food; where uneasiness, distress, or absolute pain is pretty regularly experienced, though the quantity of food be small, and the quality not unwholesome, there is a heightened condition of the organic sensibility dependent not on a state of irritation merely, but on actual inflammation; subacute, however, in degree, chronic in duration; and therefore subacuto-chronic. And if, in addition, nausea supervenes, and finally vomiting, there is reason to suspect not merely the presence of an inflammatory congestion, but of an absolute organic change; a suspicion strengthened at this time by the gradual diminution of the blood, and the decided loss of flesh, constituting a condition of universal anæmia and atrophy." 79.

In this passage it would appear to us that Dr. B. has confounded two stages of the complaint—the first stage, irritation and morbid sensibility of the stomach, with the second, where inflammatory action supervenes on irritation.

The cause of pyrosis and gastrodynia is considered by Dr. Burne as being, in a great majority of cases, "habitual constipation."

"*The Treatment* which I have pursued and found successful,—not only in relieving but in curing gastrodynia and pyrosis,—has been based on the pathological views just set forth; and conducted on the principle of restoring the natural function of the bowels,—of appeasing the irritability of the stomach,—of subduing the subacute inflammatory action,—and of removing, as far as possible, its consequences on the tissues of the stomach. The means adopted have been *calomel, opium, the local abstraction of blood, counter-irritation, and appropriate aperients.*" 85.

We need hardly remark that this would be the treatment pursued, if any other theory had been adopted. It is just as appropriate for chronic hepatitis, as for the pyrosis, but then chronic hepatitis would itself be put down by Dr. Burne as caused by "habitual constipation."

"The calomel has been given in the dose of *half a grain* and the opium of *a quarter of a grain,* twice a day; namely, after breakfast, and after dinner. At these periods the opium reconciles the stomach to the presence of food, and the calomel acts gradually as a mercurial. The dose of mercury is small in order that it might not salivate too soon; time being required to remove diseases of long standing. The effect of this combination is most happy: and success attends the treatment of almost every case which admits of recovery. The medicines are persevered in for many weeks; unless the gums become affected, when the calomel is omitted, but the opium continued. Opium was a favourite remedy of Cullen, has been adopted by others, and is an essential part of the treatment: though, when given alone, it is only palliative, as candidly stated by Cullen." 85.

Chap. VI. presents us with a frightful catalogue—"the consequences of habitual constipation." "Hyperæsthesia and hypertrophy of the womb, dysmenorrhœa, menorrhagia, amenorrhœa, leucorrhœa, abortion, miscarriage, supposed pregnancy"—these are among the effects of constipation of the bowels! It is but justice to the author to say, that he does not allot constipation as the sole and invariable cause of the above maladies; but that it ought "to be recognized as one very frequent—*perhaps the most frequent cause.*" Sir Charles Clarke recognised it as "a coexisting symptom requiring attention in the treatment," but never dreamt of regarding it as a frequent cause of such various maladies. It is needless for us to go over the list of cases brought forward by our author, as in all of them "constipation" was discovered to exist, or to have existed—and, as a matter of course, it was selected as the *cause.* But the moment the reasoning ceases, Dr. B. dismounts from his HOBBY, and treats each case exactly as any judicious and observant practitioner would do, leaving his little hobby to browse at will on the neighbouring common, till he whistles for him again.

Chap. VII.—To this chapter we have no objection, either in theory or practice. It treats of those complaints which are regarded by all practitioners as frequently caused by neglected attention to the bowels. These are colic, diarrhœa, dysentery (?), ileus, accumulation of fæces, obstruction, displacement, inflammation, ulceration, hypertrophy with induration or contraction, stricture. The following short case we shall quote. We fear the mistake that took place at Leamington, occurs every day in other places!

"A gentleman about 35 years of age, of spare habit and nervous temperament, was harassed by an irregular state of the bowels, they being generally much confined. At times he would be affected with colic pains and frequent inclination to retire to the closet, the dejections being scybalous, scanty, and mucous; which nevertheless impressed him with the belief that his bowels were absolutely relaxed; the contrary in fact being the case. His health being weak and disordered, he set out on an excursion to Leamington, and, while on the road, the colic pains increased so considerably that it was with difficulty he reached the end of his journey,

Arrived at the hotel, the pains were become most violent, and the gentleman excessively ill. Advice was obtained, and blood to a large amount abstracted under the notion of inflammation; medicines also were administered without relief. The pain persisted in all its intensity, and extinguished life in the course of sixteen hours. The body was opened and the colon discovered to be loaded with hardened feces; without any evidence or trace of inflammation." 110.

Another case is given where a fainting fit, from colic, was mistaken for epilepsy!! In the following passage we entirely agree with Dr. Burne.

"No greater error in medicine can be committed than the relying upon a single sign as indicative of a disease, which, when present, is evidenced by several. Inflammation is manifested not by pain alone, but by other signs co-existing; and therefore, when these other signs are not present, it may safely be concluded that the pain does not result from inflammation, but from spasm, or neuralgia, &c.

Remarks of a similar tendency apply to the cases of syncope, and to the possibility of mistaking them for epilepsy. Syncope is a sudden seizure from an accidental cause. Epilepsy has always premonitory and precursory signs; and, besides, the fit itself is so different from syncope, that it is only to recollect that epilepsy is attended with convulsions, foaming at the mouth, and congestion of the face and head, in order to distinguish it most readily from syncope, where there is a death-like stillness and pallor." 113.

CHAP. VIII. Is on "diseases of the rectum as consequences of habitual constipation."

This chapter requires no particular notice. We see nothing objectionable; and many of the cases detailed by the author or contributed by his friends are important. The following case from Dr. Roots, contains no bad satire on HOBBIES. Sat verbum.

"A medical practitioner under my care for general indisposition, which may not improperly be called 'a breaking up of the constitution,' from long-continued sensuality, happened to dine at the house of a friend who had been under treatment for a stricture. This friend had derived so much benefit that he strongly advised the practitioner to consult *his* surgeon. He did so; and his complaints were at once pronounced to arise from a stricture. All this he told me when I next saw him, expressing at the same time his satisfaction and belief that his disease had been discovered. 'Well,' said I, 'this I cannot understand! You have never had symptoms of stricture, for your bowels have acted regularly every day. Let the surgeon meet me at your house, and, if there is stricture, it will be obvious to me as well as to him.' We did meet. The surgeon produced a long rectum bougie, twisted it like a corkscrew, and examined the rectum; but no evidence of stricture could he afford me. The surgeon, however, persisted in his opinion that a stricture was there; and affirmed, moreover, that he could distinguish the presence of a stricture ten or twelve inches up the gut by means of the bougie. Of this I was sceptical: and left the case in his hands. The bougie treatment was persevered in, and the patient in high spirits at the prospect of cure.

In a few weeks I was requested to see the patient again. The imagined relief from the bougie had ceased with the novelty; and finding, in sad reality, that he grew worse instead of better, and remembering that he had never experienced any difficulty in the action of the bowels, he had now persuaded himself that he had *not* a stricture, and had, accordingly, declined any further mechanical treatment. He died, soon afterwards, of a sudden and severe cerebral attack; and permission having been granted to examine the body, I apprised the surgeon of it, and requested his assistance. No stricture nor trace of disease could be discovered, either in the rectum, colon, or intestinal canal." 150.

Chap. IX. Treats of "cutaneous diseases as consequences of habitual constipation," and embraces the consideration of erysipelas, erythema nodosum, psoriasis, impetigo, and acne. Our author does not pretend that all kinds of erysipelas result from constipation, but only one particular kind, seldom attended by danger, and generally affecting one side of the face. It is gradual in progress—less intensely red, and hot, and hard than phlegmonoid erysipelas, unaccompanied by pyrexia.

Chap. X. We now come to the subject which stands foremost in the title-page of the work—the *causes* of constipation. Inattention to the calls of nature, so prevalent among all classes, but especially among females, is placed in the first rank by Dr. Burne. The inconvenience or want of comfortable water-closets is strongly dwelt on by our author—particularly on the Continent. The "lieux d'aisance," however, are much better regulated now than formerly, among all the hotels frequented by the English.

*Civilization and refinement* contribute not a little to "habitual constipation," by overworking the brain and underworking the limbs. Grief and anxiety have a peculiar influence over the bowels, producing constipation in the majority; but relaxation in some individuals.

"The anxiety of mind too arising out of an advanced state of civilisation has a great influence on the functions of the alimentary canal; not merely in causing indigestion, but in rendering torpid the peristaltic action of the intestines. Look at the multitude of anxious faces one meets daily in the streets. Think for a moment on the contentions, competitions, and responsibilities that men have to encounter, and we shall not be surprised at the load of anxiety that oppresses the greater part. This anxiety depresses the energy of the nervous system and of all the organic functions. The powers of the stomach become languid, and digestion is imperfect; the liver becomes sluggish and the supply of bile is defective; and the peristaltic action of the intestinal canal grows slow and retards the excretions: and thus do affections of the mind produce languor, indigestion, constipated bowels, and all their consequences." 167.

The sedentary habits of females, artisans, lawyers, merchants and various other classes, strongly conduce to constipation of the bowels. Travelling by sea or land usually induces costiveness; but this kind of constipation seldom produces any mischief, the exercise and change of air counterbalancing the inconvenience of costive bowels. Dr. B. runs over the pathological and mechanical causes, including inflammation, spasm, or other affections of the alimentary canal—laxity or debility of the muscular fibres—torpor of the liver and paucity of bile—diseases of the brain—abnormal secretion or discharges from other parts of the body diminishing the succus intestinalis, as in diabetes for example. Then the mechanical causes may depend on stricture of some part of the canal—organised bands crossing the gut—accidental adhesions or displacements. The accumulation of balls in the bowels—as of magnesia, chalk, &c. The carbonate of iron in our own practice, has been found blocking up the rectum, and causing great distress. Dr. Paris found balls of cubebs in the bowels—and the white mustard seed is notorious for forming these accumulations. The egg-cup found by Mr. Dendy in the ileum of a man is amongst the most extraordinary—perhaps, however, not more so than the knives found in the stomach and bowels of a knife-eater.

CHAP. XI. is on the treatment of constipation. There are various means besides merely aperient medicines, which tend to counteract this troublesome disposition. Mild means, in such cases, are generally better than rough remedies. Dr. B. relates a case of obstinate constipation cured by goat's whey in Switzerland. Although as a general rule, the bowels ought to be opened once in twenty-four hours; yet there are numerous exceptions, where people in health have three or four motions daily, whilst others require only one evacuation every three, four, or five days. These peculiarities should always be taken into consideration.

*Regimen.*—Early rising favours regularity of bowels. About eight hours for rest, or one third of our existence in bed, is a normal quantity of sleep—though few can pass all that time in a state of temporary annihilation.

"It is the lingering in bed, the going to sleep a second time after having enjoyed a good night's rest, that does the mischief. A person awakes refreshed, light, cheerful: but if, instead of at once getting up, he dozes off to sleep again, he afterwards rises with unwillingness; and finds his head heavy, his spirits dull, and his bowels indisposed to act." 197.

*Habit.*—We are creatures of habit, and we know many people who, by dint of habitual application to the temple of Cloacina for relief, have at length obtained it permanently.

*Exercise* immediately after breakfast is beneficial; but alas! how few are able to devote the forenoon hours to anything but business or study! A change from town to country, or even *vice versa*, will sometimes produce a change in the action of the bowels.

*Diet*, however, is the main counter-acter of constipation. A glass of cold water early in the morning has often given a stimulus to the bowels. Coarse brown bread often suffices for this purpose. Figs, prunes, mustard seed, ripe fruits and vegetables have been advantageously employed by many people. Boiled or broiled bacon for breakfast is a popular remedy, and not without considerable effect.

“Like the other articles which have been enumerated, bacon, particularly broiled, should not be taken daily for too long a period, or it may recoil upon and satiate the stomach. It is better to vary diet from time to time, and to endeavour, by the judicious and alternate use of the several means described, to bring about and retain the natural function of the intestinal canal. Trivial and unimportant as these means may appear, they nevertheless exercise a decided and beneficial influence, and will often go far to re-establish the health, which has been much disordered by habitual constipation.” 200.

*Aperient Medicines.*—Dietetics will often fail, and the doctor is applied to at last. A great number of patients appeal to this personage for the means of keeping their bowels open without having recourse to medicine at all—and where they will take very small pains themselves in respect to diet. Dr. B. justly observes, that the restitution of function in the alimentary canal is not to be obtained by *purgative* medicines, which too often leave the bowels more torpid than before their operation. But we have not found it so easy a matter to find other aperients—“which operate equally well and leave the bowels disposed to their natural functions.” This would be, indeed, “a desideratum of the first importance.”

“The medicines prescribed are pretty uniformly the same; colocynth and calomel, or a ‘black dose’ of senna and salts, being the ordinary resource. They purge the bowels but distress the patient; and leave him equally or more liable to constipation.

In speaking thus, let me not be understood as speaking too generally: the exceptions to the remark are many, but it will, I apprehend, be admitted that the system of giving single aperient or cathartic doses, rather than of pursuing a systematic course of laxative treatment, prevails.” 202.

Aperients must vary according to the age as well as the temperament of individuals. In childhood, castor oil and senna are generally proper, where there is merely constipation, unattended by any of those disorders which arise from the same causes that produce constipation. In advanced life the more active and resinous aperients are necessary, as colocynth, aloes, gamboge, scammony, &c. Aperients must also vary on account of accidental local affections, as piles, irritation of bladder, uterus, or kidneys, &c. For habitual constipation, unaccompanied by other disorder, there are few things better than a dinner pill of from four to ten grains of the pil. rhei comp.—or four or five grains of the root of rhubarb chewed before dinner. Dr. B. gives us a running commentary on the various individual aperients, containing judicious remarks that will be very useful to the non-professional invalid, but which need not be noticed here. He has a section on the bougie—lavements—and suppositories. Dr. B. thinks, and we agree with him, that the bougie might be much more frequently used than it is at present. Putting aside the quackeries of the rectum doctors, we have seen very excellent effects result from the bougie, where there was tendency to stricture, whether spasmodic or organic, of the gut. The dilatation of the rectum seems to act by sympathy on the whole line of the colon, and often to produce an evacuation, which would not otherwise take place. The irritable sphincter ani, a complaint by no means uncommon, is greatly relieved by the bougie.

With Dr. Burne's observations on lavements we cannot quite agree. From personal and general observations of thirty years, we can speak favourably of these means, and confidently.

" As a means of obviating habitual constipation they are employed by some persons daily, with the effect of exonerating the large intestine more or less. However efficient they may, in this respect, appear to be, I have found, from long observation, that they are not free from very great objection. In the first place, they do not continue to relieve the bowels fully and freely for any length of time:—in the next place, they do not dispose the bowels to resume their natural action, but, on the contrary, render them more confined:—in the third place, they wash off the mucus from the intestine, which is followed by a degree of irritation and an unpleasant sense of heat, very similar to that which occurs after washing the hands in water simply:—in the fourth place, the fæces become more scybalous and hard under their use:—and lastly, the individual does not feel the comfort or conviction of having had his bowels fully relieved; on which account he is often induced to resort to a second lavement on the same day. Lavements fail in completely obviating or curing habitual constipation." 230.

From experience much longer, and, we apprehend, much wider than Dr. Burne could possibly have, we hesitate not to aver that most of the above objections are greatly magnified, and some of them are of such rare occurrence as to be quite unworthy of notice. How is it that the French, Germans, and other continental people use lavements all their lives, without these inconveniences, and with the happy effect of saving themselves oceans of draughts and bushels of pills? We have not found the fæces become " hard and scybalous " from the use of injections—nor will the mucus of the bowels be washed away by warm water, which ought always to be used. Few people in this country, at least, know the best mode of employing lavements. The operation should take place either in the water-closet, or in the bed-room with the commode at hand. The pump should be plied till a strong nisus for evacuation occurs, whatever be the quantity necessary. Then there is a quick, copious, and complete clearance of the colon. The throwing up of a pint or even two pints into the bowels, waiting for the nisus a quarter or half an hour, is quite unnecessary and not so effectual as the plan related. Even the continentals are very mistaken on this point. They think they cannot retain the lavement too long—and the consequence is that, not very unfrequently, it is not thrown off at all. The bowel should be distended till the contraction commences.

" Lavements are nevertheless an excellent *occasional* resource; where, for example, a person has been disappointed of his usual evacuation, or where he is unable, in consequence of his engagements or of other circumstances, to take an habitual aperient, lest it may act at an inconvenient moment. In this case a lavement used in the morning relieves the bowels for the day, and is a great comfort. Or if, while travelling, the bowels become unusually constipated, and yet it is not convenient or scarcely possible to venture on medicine sufficiently strong to act, then a lavement is of essential service, and often prevents absolute indisposition, as I have myself witnessed." 230.

This passage goes far to neutralize the tendency of the preceding one. Dr. B. objects to warm water as an enema. Cold water injected into the bowels would, in two cases out of three, produce disagreeable colic, and not act properly after all.

The last or twelfth chapter is a kind of resumé or analysis of Mr. Annesley's "Observations on the causes, nature, and treatment of the more prevalent Diseases of India," of which excellent work we have inserted numerous notices in this Journal.

As we have said more than once in this article, we find little or no fault with Dr. Burne's practice in the various diseases enumerated. On the contrary, we think it judicious and discriminating. But we must be excused for demurring to his *theory*. We cannot coerce our opinions or rather convictions on that point, and we state our objections with every possible respect for the talents and acquirements of the Author.

